# Supplementary material for: Comparative Study on the Structural Properties and Bioactivities of Three Different Molecular Weights of Lycium barbarum Polysaccharides
Source: Molecules. 2023 Jan 10;28(2):701. doi: 10.3390/molecules28020701 (PMC9867462; doi:10.3390/molecules28020701)
Supplement: Supplementary file 1 [file molecules-28-00701-s001.zip › molecules-2073533-supplementary.pdf]

Supplementary Materials for

Comparative Study on the Structural Properties and Bioactivities of Three Different Molecular Weights of *Lycium barbarum* Polysaccharides

Wenjun Zeng [1,2], Lulu Chen [1,2], Zhihui Xiao [3], Yanping Li [4], Jianlong Ma [5,6], Jianbao Ding [4,*] and Jin Yang [1,2,5,*]

[1] School of Chemistry and Chemical Engineering, North Minzu University, Yinchuan 750021, China
[2] Key Laboratory for Chemical Engineering and Technology, North Minzu University, State Ethnic Affairs Commission, Yinchuan 750021, China
[3] South China Sea Institute of Oceanology, Chinese Academy of Sciences, Guangzhou 510301, China
[4] Ningxia Wuxing Science and Technology Co., Ltd., Yinchuan 750021, China
[5] Ningxia Research Center for Natural Medicine Engineering and Technology, Yinchuan 750021, China
[6] College of Chemistry and Chemical Engineering, Ningxia University, Yinchuan 750021, China
* Correspondence: nxwxdjb@126.com (J.D.); yang_jin@nun.edu.cn (J.Y.); Tel.: +86-951-6048881 (J.D.); +86-951-2067917 (J.Y.)

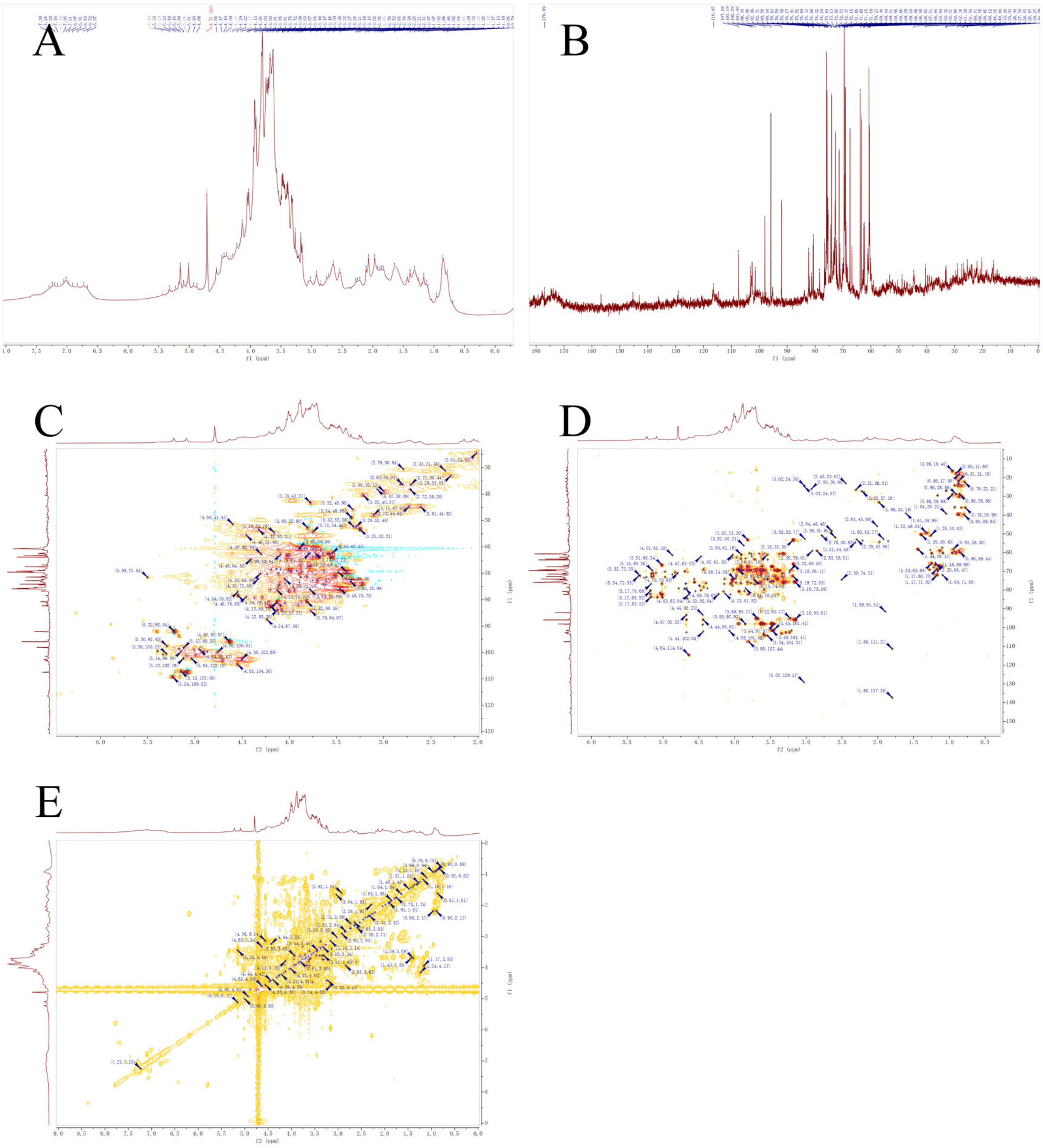

Figure S1. NMR spectra of LBPs-2: ^{1}H NMR spectra (D_2O, 700 MHz) (**A**); ^{13}C NMR spectra (D_2O, 175 MHz) (**B**); HSQC spectra (**C**); HMBC spectra (**D**); ^{1}H-^{1}H COSY spectra (**E**).

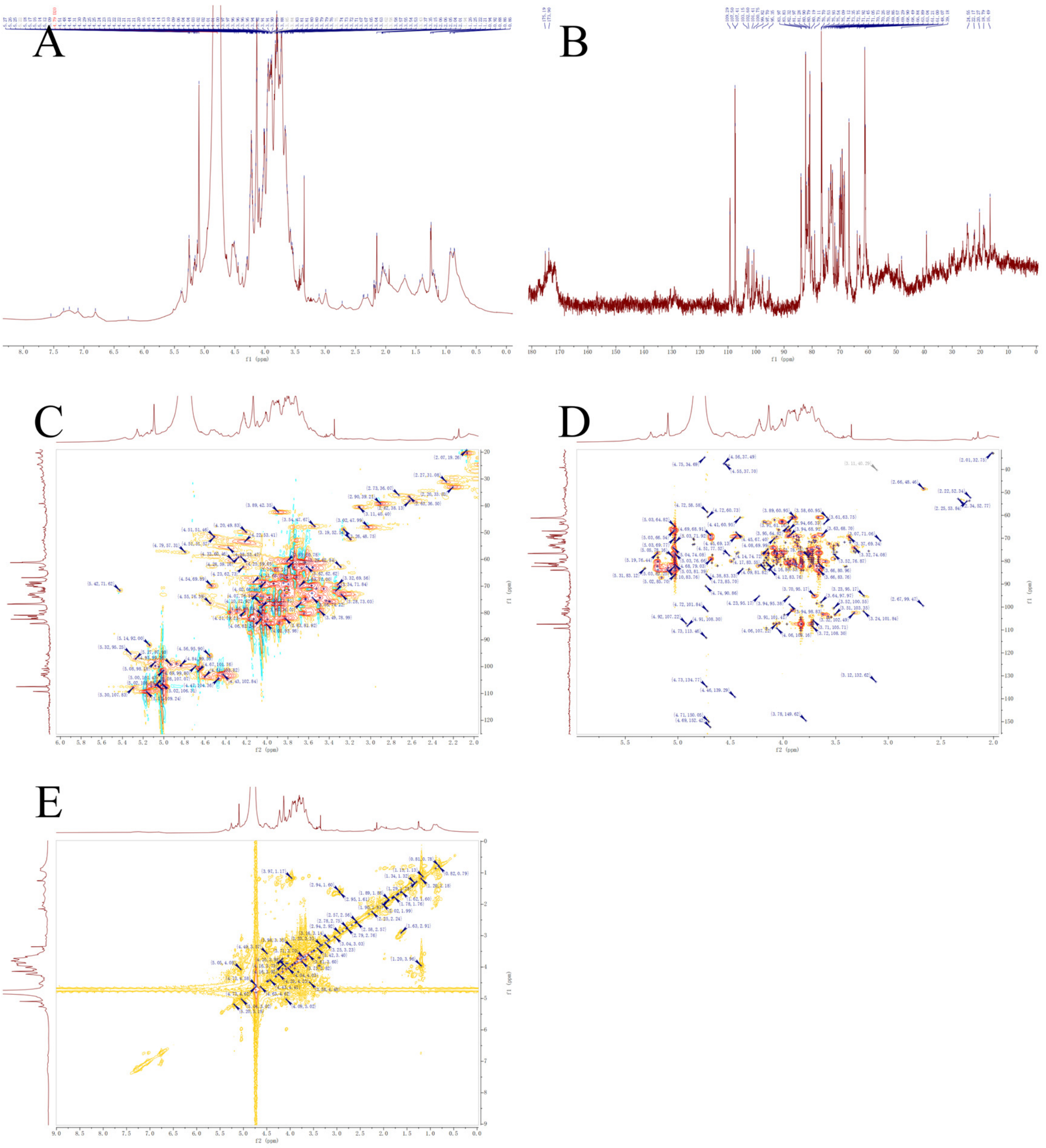

Figure S2. NMR spectra of LBPs-3: ^{1}H NMR spectra (D_2O, 700 MHz) (**A**); ^{13}C NMR spectra (D_2O, 175 MHz) (**B**); HSQC spectra (**C**); HMBC spectra (**D**); ^{1}H-^{1}H COSY spectra (**E**).
